# Lyapunov-type inequalities for quasilinear elliptic equations with Robin boundary condition

**DOI:** 10.1186/s13660-017-1320-4

**Published:** 2017-02-16

**Authors:** Ülkü Dinlemez Kantar, Tülay Özden

**Affiliations:** 1 Department of Mathematics, Faculty of Sciences, Gazi University, 06500 Teknikokullar, Ankara, Turkey; 2 Department of Mathematics, Institute of Sciences, Gazi University, 06500 Teknikokullar, Ankara, Turkey

**Keywords:** 35J40, 35J66, 46E35, Lyapunov-type inequalities, elliptic equations, Robin boundary conditions

## Abstract

The aim of this study is to prove Lyapunov-type inequalities for a quasilinear elliptic equation in $\mathbb{R}^{2}$. Also the lower bound for the first positive eigenvalue of the boundary value problem is obtained.

## Introduction

In [1], Lyapunov proved that, if $p(x)$ is a nonnegative and continuous function and $u(x)\in C(I,\mathbb{R})$, a necessary condition for the following boundary value problem:

1.1 $$ \left \{ \textstyle\begin{array}{l}u^{\prime\prime}(x)+p(x)u(x)=0, \quad u(x)\neq0,\forall x\in I, \\ u(a_{1})=0=u(b_{1}), \end{array}\displaystyle \right . $$

to have nontrivial solutions is

1.2 $$ 4/(b_{1}-a_{1})\leq \int_{a_{1}}^{b_{1}}p(x)\,\mathrm{d}x, $$

where $I= [ a_{1},b_{1} ] $.

Since Lyapunov’s study, because the inequality of (1.2) plays a key role for the qualitative properties, such as oscillatory and disconjugacy etc., of differential equations’ solutions, several authors focused on the inequality of (1.2). Those authors improved and generalized the inequality of (1.2) in $\mathbb{R}$. In this work the literature of the one-dimensional case is not studied in detail but it is listed in the references for the interested reader. See [1–17] and the references cited therein.

In addition to studies in $\mathbb{R}$, several authors [18–24] have extended the inequality of (1.2) in $\mathbb{R}^{n}$ recently. To the best of our knowledge, it was extended by Cañada, Montero, and Villegas [19] for the first time. In [19] Cañada *et al.* considered the linear elliptic problem as follows:

1.3 $$ \left \{ \textstyle\begin{array}{l}-\triangle u=a(x)u,\quad x\in\Omega, \\ \partial u/\partial n=0,\quad x\in\partial\Omega, \end{array}\displaystyle \right . $$

where $\Omega\subset \mathbb{R}^{N}$ is a smooth bounded domain with $N\geq2$ and the function $a:\Omega \rightarrow \mathbb{R}$ belongs to the set

1.4 $$ \Lambda= \biggl\{ a\in L^{N/2}(\Omega)\setminus\{0\}: \int_{\Omega}a(x)\,\mathrm{d}x\geq0\text{ and (1.3) has a nontrivial solution} \biggr\} $$

if $N\geq3$,

1.5 $$\begin{aligned} \Lambda =&\biggl\{ a:\Omega\rightarrow \mathbb{R} \text{ s.t. } \exists q\in( 1,\infty ] \text{ with }a\in L^{q}(\Omega)\setminus \{0\}: \int_{\Omega}a(x)\,\mathrm{d}x\geq0 \\ &\text{and (1.3) has a nontrivial solution}\biggr\} \end{aligned}$$

if $N=2$, we define

1.6 $$ \beta_{q}:=\inf_{a\in\wedge\cap L^{q}(\Omega)}\Vert a\Vert _{L^{q}(\Omega)}, \quad 1\leq q\leq\infty. $$

Their main result is as follows.

### Theorem A

*The following statements hold*.

1. *If* $N=2$ *then* $\beta_{q}>0 \Leftrightarrow 1< q\leq\infty$. *If* $N\geq3$ *then* $\beta _{q}>0 \Leftrightarrow\frac{N}{2}\leq q\leq\infty$.
2. *If* $\frac{N}{2}< q\leq\infty$ *then* $\beta_{q}$ *is attained*. *In this case*, *any function* $a\in\wedge\cap L^{q}(\Omega)$ *from which* $\beta_{q}$ *is attained has one of the following forms*:
  (i) $a(x)=\lambda_{1}$, *if* $p=\infty$, *where* $\lambda_{1}$ *is the first strictly positive eigenvalue of* (1.3).
  (ii) $a(x)=\vert u(x)\vert ^{{\frac{2}{p-1}}}$, *if* $\frac{N}{2}< q<\infty$, *where* *u* *is a solution of the problem as follows*:
    1.7 $$ \left \{ \textstyle\begin{array}{l}-\triangle u=\vert u(x)\vert ^{2/(p-1)}u, \quad x\in\Omega, \\ \partial u/\partial n=0, \quad x\in\partial\Omega. \end{array}\displaystyle \right . $$
3. *The map* $(\frac{N}{2},\infty)\rightarrow \mathbb{R}$, $p\mapsto\beta_{p}$, *is continuous and the map* $[\frac{N}{2},\infty ) \rightarrow \mathbb{R}$, $p\mapsto \vert \Omega \vert ^{\frac{-1}{p}}\beta_{p}$, *is strictly increasing*.
4. *The limits* $\lim_{p\rightarrow\infty}\beta_{p}$ *and* $\lim_{p\rightarrow(\frac{N}{2})^{+}}\beta_{p}$ *always exist and take the values*
  (i) $\lim_{p\rightarrow\infty}\beta_{p}=\beta_{\infty}$, *if* $N\geq2$,
  (ii) $\lim_{p\rightarrow(\frac{N}{2})^{+}}\beta _{p}\geq \beta_{\frac{N}{2}}>0$, *if* $N\geq3$, $\lim_{p\rightarrow1^{+}}\beta_{p}=0$, *if* $N=2$.

Here, we also note that in the study Cañada *et al.* they proved that the relation between the *p* and $\frac{N}{2}$ plays a crucial role. They also considered the equation in (1.3) with zero Dirichlet boundary condition. They presented similar inequalities at their study. Then others established Lyapunov-type inequalities for different equations with boundary conditions. For more information about the studies in $\mathbb{R}^{n}$, the interested reader can refer to [18–24] and the references cited therein.

The aim of this paper is to prove a Lyapunov-type inequality for the two-dimensional quasilinear elliptic problem as follows:

1.8 $$ \left \{ \textstyle\begin{array}{l}-(\Phi_{p}(u_{xy}))_{y}=r(x,y)\Phi_{p}(u),\quad (x,y)\in\Omega, \\ u(a_{1},y)=0=u(b_{1},y), \quad a_{2}\leq y\leq b_{2}, \\ u_{x}(x,a_{2})=0=u_{x}(x,b_{2}),\quad a_{1}\leq x\leq b_{1}, \end{array}\displaystyle \right . $$

where $\Omega= [ a_{1},b_{1} ] \times [ a_{2},b_{2} ] $ and $r(x,y)$ is a measurable function on Ω, and $\Phi_{p}(u(x,y))=\vert u(x,y)\vert ^{p-2}u(x,y)$ for $p>1$. In addition to this, we note that by a solution of the problem (1.8), we mean that $u(x,y)\in W^{3,p}(\Omega)$ in that

1.9 $$ W^{3,p}(\Omega)= \bigl\{ u: u,u_{x},u_{xy}\text{ and }u_{xyy}\in L^{p}(\Omega) \bigr\} . $$

As usual, $L^{p}(\Omega)$ is a space of Lebesgue measurable functions.

## Main results

Now, we give a key lemma as a proof of our main conclusions.

### Lemma 1

*Assume that* $u(x,y)\in W^{3,p}(\Omega )$, *it satisfies the boundary conditions in* (1.8) *and* $u(x,y)\neq0$ *for* $\forall(x,y)\in\Omega^{0}$. *Then*

2.1 $$\begin{aligned}& \bigl(4\bigl\vert u(x,y)\bigr\vert \bigr)^{p}/(b_{1}-a_{1})^{p-1}(b_{2}-a_{2})^{p-1} \leq { \int_{a_{1}}^{b_{1}}} { \int_{a_{2}}^{b_{2}}} \vert u_{xy}\vert ^{p}\,\mathrm{d}y\,\mathrm{d}x, \end{aligned}$$

2.2 $$\begin{aligned}& \bigl( 2^{p}/(b_{2}-a_{2})^{p-1} \bigr) { \int_{a_{1}}^{b_{1}}} \vert u_{x}\vert ^{p}\,\mathrm{d}x\leq { \int_{a_{1}}^{b_{1}}} { \int_{a_{2}}^{b_{2}}} \vert u_{xy}\vert ^{p}\,\mathrm{d}y\,\mathrm{d}x, \end{aligned}$$

*hold*, *respectively*, *where* $\Omega^{0}$ *is the set of all interior points of* Ω.

### Proof

Let $(x,y)\in\Omega$. Since $u(x,y)$ satisfies the boundary conditions in (1.8), it is easy to see

$$u(x,y)= { \int_{a_{1}}^{x}} { \int_{a_{2}}^{y}} u_{ts}\,\mathrm{d}s\, \mathrm{d}t, $$

taking the absolute value, we obtain

2.3 $$ \bigl\vert u(x,y)\bigr\vert \leq { \int_{a_{1}}^{x}} { \int_{a_{2}}^{y}} \vert u_{ts}\vert \, \mathrm{d}s\,\mathrm{d}t. $$

Similarly, we get

2.4 $$\begin{aligned}& \bigl\vert u(x,y)\bigr\vert \leq { \int_{a_{1}}^{x}} { \int_{y}^{b_{2}}} \vert u_{ts}\vert \, \mathrm{d}s\,\mathrm{d}t, \end{aligned}$$

2.5 $$\begin{aligned}& \bigl\vert u(x,y)\bigr\vert \leq { \int_{x}^{b_{1}}} { \int_{a_{2}}^{y}} \vert u_{ts}\vert \, \mathrm{d}s\,\mathrm{d}t, \end{aligned}$$

and

2.6 $$ \bigl\vert u(x,y)\bigr\vert \leq { \int_{x}^{b_{1}}} { \int_{y}^{b_{2}}} \vert u_{ts}\vert \, \mathrm{d}s\,\mathrm{d}t. $$

Adding (2.3)-(2.6), we have

2.7 $$ 4\bigl\vert u(x,y)\bigr\vert \leq { \int_{a_{1}}^{b_{1}}} { \int_{a_{2}}^{b_{2}}} \vert u_{xy}\vert \, \mathrm{d}y\,\mathrm{d}x. $$

Then, applying Hölder’s inequality

2.8 $$ \int_{a_{1}}^{b_{1}}\bigl\vert f(t)g(t)\bigr\vert \, \mathrm{d}t\leq \biggl( \int_{a_{1}}^{b_{1}}\bigl\vert f(t)\bigr\vert ^{q}\,\mathrm{d}t \biggr) ^{1/q} \biggl( \int_{a_{1}}^{b_{1}}\bigl\vert g(t)\bigr\vert ^{p}\,\mathrm{d}t \biggr) ^{1/p} $$

to the right-hand side of (2.7), we get

2.9 $$ \bigl( 4\bigl\vert u(x,y)\bigr\vert \bigr) ^{p}\leq ( b_{1}-a_{1} ) ^{p-1}{ \int_{a_{1}}^{b_{1}}} \biggl[ { \int_{a_{2}}^{b_{2}}} \vert u_{xy}\vert \, \mathrm{d}y \biggr] ^{p}\,\mathrm{d}x. $$

Applying Hölder’s inequality to the right hand side of (2.9) again, we obtain

$$\frac{(4\vert u(x,y)\vert )^{p}}{(b_{1}-a_{1})^{p-1}(b_{2}-a_{2})^{p-1}}\leq { \int_{a_{1}}^{b_{1}}} { \int_{a_{2}}^{b_{2}}} \vert u_{xy}\vert ^{p}\,\mathrm{d}y\,\mathrm{d}x. $$

Thereby, the proof of (2.1) is completed.

Similarly we have

2.10 $$ \bigl\vert u_{x}(x,y)\bigr\vert \leq { \int_{a_{2}}^{y}} \vert u_{xs}\vert \, \mathrm{d}s $$

and

2.11 $$ \bigl\vert u_{x}(x,y)\bigr\vert \leq { \int_{y}^{b_{2}}} \vert u_{xs}\vert \, \mathrm{d}s. $$

Adding (2.10) and (2.11), we get

2.12 $$ 2\bigl\vert u_{x}(x,y)\bigr\vert \leq { \int_{a_{2}}^{b_{2}}} \vert u_{xs}\vert \, \mathrm{d}s. $$

Applying Hölder’s inequality to the right hand side of (2.12) and integrating from $a_{1}$ to $b_{1}$, we have

$$\frac{2^{p}}{(b_{2}-a_{2})^{p-1}}{ \int_{a_{1}}^{b_{1}}} \vert u_{x}\vert ^{p}\,\mathrm{d}x\leq { \int_{a_{1}}^{b_{1}}} { \int_{a_{2}}^{b_{2}}} \vert u_{xy}\vert ^{p}\,\mathrm{d}y\,\mathrm{d}x. $$

Consequently, the proof of (2.2) is completed. □

### Theorem 1

*If* $u(x,y)\in W^{3,p}(\Omega )$ *is a nontrivial solution of the problem* (1.8), *then the following inequality*:

2.13 $$ 2^{2p+1}/(b_{1}-a_{1})^{p-1}(b_{2}-a_{2})^{p} \leq { \int_{a_{1}}^{b_{1}}} { \int_{a_{2}}^{b_{2}}} \bigl\vert r(x,y)\bigr\vert ^{q}\,\mathrm{d}y\,\mathrm{d}x $$

*holds*, *where* *q* *is the Hölder conjugate of* *p*.

### Proof

Let $u(x,y)\in W^{3,p}(\Omega)$ is a nontrivial solution of the problem (1.8). Multiplying the equation in (1.8) by $u_{x}$ and integrating on Ω,we obtain

2.14 $$ { \int_{a_{1}}^{b_{1}}} { \int_{a_{2}}^{b_{2}}} -\bigl(\vert u_{xy} \vert ^{p-2}u_{xy}\bigr)_{y}u_{x}\, \mathrm{d}y\,\mathrm{d}x= { \int_{a_{1}}^{b_{1}}} { \int_{a_{2}}^{b_{2}}} r(x,y)\vert u\vert ^{p-2}uu_{x}\,\mathrm{d}y\,\mathrm{d}x. $$

Then, applying partial integration in ${ \int_{a_{2}}^{b_{2}}} -(\vert u_{xy}\vert ^{p-2}u_{xy})_{y}u_{x}\, \mbox{d}y$ and using the boundary conditions in (1.8), we have

2.15 $$ { \int_{a_{1}}^{b_{1}}} { \int_{a_{2}}^{b_{2}}} \vert u_{xy}\vert ^{p}\,\mathrm{d}y\,\mathrm{d}x={ \int_{a_{1}}^{b_{1}}} { \int_{a_{2}}^{b_{2}}} r(x,y)\vert u\vert ^{p-2}uu_{x}\,\mathrm{d}y\,\mathrm{d}x. $$

By taking the absolute value on right hand side of (2.15), we get

2.16 $$ { \int_{a_{1}}^{b_{1}}} { \int_{a_{2}}^{b_{2}}} \vert u_{xy}\vert ^{p}\,\mathrm{d}y\,\mathrm{d}x\leq { \int_{a_{1}}^{b_{1}}} { \int_{a_{2}}^{b_{2}}} \bigl\vert r(x,y)\bigr\vert \vert u\vert ^{p-1}\vert u_{x}\vert \,\mathrm{d}y\, \mathrm{d}x. $$

Hence, applying Hölder’s inequality to the right hand side of (2.16), we find

2.17 $$\begin{aligned}& { \int_{a_{1}}^{b_{1}}} { \int_{a_{2}}^{b_{2}}} \vert u_{xy}\vert ^{p}\,\mathrm{d}y\,\mathrm{d}x \\& \quad \leq \biggl( { \int_{a_{1}}^{b_{1}}} { \int_{a_{2}}^{b_{2}}} \bigl\vert r(x,y)\bigr\vert ^{q}\vert u\vert ^{(p-1)q}\,\mathrm{d}y\, \mathrm{d}x \biggr) ^{1/q} \biggl( { \int_{a_{1}}^{b_{1}}} { \int_{a_{2}}^{b_{2}}} \vert u_{x}\vert ^{p}\,\mathrm{d}y\,\mathrm{d}x \biggr) ^{1/p}. \end{aligned}$$

Now, considering only the second term of right hand side in (2.17) from Fubini’s theorem, we can rewrite the inequality (2.17) as follows:

2.18 $$\begin{aligned}& { \int_{a_{1}}^{b_{1}}} { \int_{a_{2}}^{b_{2}}} \vert u_{xy}\vert ^{p}\,\mathrm{d}y\,\mathrm{d}x \\& \quad \leq \biggl( { \int_{a_{1}}^{b_{1}}} { \int_{a_{2}}^{b_{2}}} \bigl\vert r(x,y)\bigr\vert ^{q}\vert u\vert ^{(p-1)q}\,\mathrm{d}y\, \mathrm{d}x \biggr) ^{1/q} \biggl( { \int_{a_{2}}^{b_{2}}} \biggl[ { \int_{a_{1}}^{b_{1}}} \vert u_{x}\vert ^{p}\,\mathrm{d}x \biggr] \,\mathrm{d}y \biggr) ^{1/p}. \end{aligned}$$

Hence, using the inequality (2.2) in (2.18), we obtain

2.19 $$\begin{aligned}& \biggl( { \int_{a_{1}}^{b_{1}}} { \int_{a_{2}}^{b_{2}}} \vert u_{xy}\vert ^{p}\,\mathrm{d}y\,\mathrm{d}x \biggr) ^{(p-1)/p} \\& \quad \leq \bigl( (b_{2}-a_{2})/2 \bigr) \biggl( { \int_{a_{1}}^{b_{1}}} { \int_{a_{2}}^{b_{2}}} \bigl\vert r(x,y)\bigr\vert ^{q}\vert u\vert ^{(p-1)q}\,\mathrm{d}y\, \mathrm{d}x \biggr) ^{1/q}. \end{aligned}$$

Then, replacing the point of $(x,y)$, which is used in Lemma 1, with the maximum point of $\vert u(x,y)\vert $, from (2.1), we get

2.20 $$\begin{aligned}& \bigl(4\max\bigl\vert u(x,y)\bigr\vert \bigr)^{(p-1)}/(b_{1}-a_{1})^{(p-1)/q}(b_{2}-a_{2})^{(p-1)/q} \\& \quad \leq \biggl( { \int_{a_{1}}^{b_{1}}} { \int_{a_{2}}^{b_{2}}} \vert u_{xy}\vert ^{p}\,\mathrm{d}y\,\mathrm{d}x \biggr) ^{(p-1)/p}. \end{aligned}$$

Then, using the inequality (2.20) in the inequality (2.19), we have

2.21 $$\begin{aligned}& \bigl(4\max\bigl\vert u(x,y)\bigr\vert \bigr)^{(p-1)}/(b_{1}-a_{1})^{(p-1)/q}(b_{2}-a_{2})^{(p-1)/q} \\& \quad \leq \bigl( (b_{2}-a_{2})/2 \bigr) \biggl( { \int_{a_{1}}^{b_{1}}} { \int_{a_{2}}^{b_{2}}} \bigl\vert r(x,y)\bigr\vert ^{q}\vert u\vert ^{(p-1)q}\,\mathrm{d}y\, \mathrm{d}x \biggr) ^{1/q} \\& \quad \leq \bigl( (b_{2}-a_{2})/2 \bigr) \biggl( { \int_{a_{1}}^{b_{1}}} { \int_{a_{2}}^{b_{2}}} \bigl\vert r(x,y)\bigr\vert ^{q}\,\mathrm{d}y\,\mathrm{d}x \biggr) ^{1/q}\bigl( \max\bigl\vert u(x,y)\bigr\vert \bigr)^{(p-1)}. \end{aligned}$$

Since $u(x,y)$ is a nontrivial solution, we have $\max \vert u(x,y)\vert \neq0$. Therefore, we obtain

2.22 $$ 2^{2p+1}/(b_{1}-a_{1})^{p-1}(b_{2}-a_{2})^{p} \leq { \int_{a_{1}}^{b_{1}}} { \int_{a_{2}}^{b_{2}}} \bigl\vert r(x,y)\bigr\vert ^{q}\,\mathrm{d}y\,\mathrm{d}x. $$

Thus, the proof is completed. □

### Corollary 1

*Let* $\lambda_{1}$ *be the first eigenvalue of the equation that is defined on* Ω *as follows*:

$$-\bigl(\Phi_{p}(u_{xy})\bigr)_{y}= \lambda_{1}r(x,y)\Phi_{p}(u), $$

*where* Ω *is a domain*, *which is defined in the beginning of the paper*, *and with the boundary conditions in* (1.8). *Then we have*

2.23 $$ 2^{2p+1}/(b_{1}-a_{1})^{p-1}(b_{2}-a_{2})^{p} \bigl\Vert r(x,y) \bigr\Vert _{L^{q}(\Omega)}^{q}\leq \lambda_{1}. $$

### Remark 1

If we take the Dirichlet boundary conditions, which are $u(a_{1},y)=0=u(b_{1},y)$ and $u(x,a_{2})=0=u(x,b_{2})$, instead of the Robin boundary conditions in the problem (1.8), then we obtain the identical conclusions given above.

### Remark 2

The result, which is obtained in this study, is also the necessary condition for the problem of (1.8) to have a nontrivial solution.

## References

[1] Lyapunov AM (1907). Problème général de la stabilité du mouvement. Ann. Fac. Sci. Univ. Toulouse.

[2] Hartman P, Wintner A (1951). On an oscillation criterion of Lyapunov. Am. J. Math..

[3] Cheng SS (1991). Lyapunov inequalities for differential and difference equations. Fasc. Math..

[4] Kwong MK (1981). On Lyapunov’s inequality for disfocality. J. Math. Anal. Appl..

[5] Panigrahi S (2009). Lyapunov-type integral inequalities for certain higher order differential equations. Electron. J. Differ. Equ..

[6] Parhi N, Panigrahi S (2003). Disfocality and Liapunov-type inequalities for third-order equations. Appl. Math. Lett..

[7] Parhi N, Panigrahi S (2002). Lyapunov-type inequality for higher order differential equations. Math. Slovaca.

[8] Parhi N, Panigrahi S (2002). Lyapunov-type inequality for delay-differential equations of third order. Czechoslov. Math. J..

[9] Parhi N, Panigrahi S (1999). On Lyapunov-type inequality for third-order differential equations. J. Math. Anal. Appl..

[10] Lee C, Yeh C, Hong C, Agarwal RP (2004). Lyapunov and Wirtinger inequalities. Appl. Math. Lett..

[11] Yang X, Kim Y, Lo K (2011). Lyapunov-type inequalities for a class of quasilinear systems. Math. Comput. Model..

[12] Yang X (2003). On inequalities of Lyapunov-type. Appl. Math. Comput..

[13] Yang X (2003). On Liapunov-type inequality for certain higher-order differential equations. Appl. Math. Comput..

[14] Pachpatte BG (1995). On Lyapunov-type inequalities for certain higher order differential equations. J. Math. Anal. Appl..

[15] He X, Tang XH (2012). Lyapunov-type inequalities for even order differential equations. Commun. Pure Appl. Anal..

[16] Youmei Z, Qingling Z, Tamaki T, Min C (2013). Admissibility for positive continuous-time descriptor systems. Int. J. Syst. Sci..

[17] Agarwal RP, Özbekler A (2015). Lyapunov type inequalities for even order differential equations with mixed nonlinearities. J. Inequal. Appl..

[18] Cañada A, Montero JA, Villegas S (2005). Lyapunov-type inequalities and applications to PDE. Elliptic and Parabolic Problems.

[19] Cañada A, Montero JA, Villegas S (2006). Lyapunov inequalities for partial differential equations. J. Funct. Anal..

[20] Hashizuma, M, Takahashi, F: Lyapunov inequality for an elliptic problem with the robin boundary condition. Preprint

[21] Timoshin SA (2010). Lyapunov inequality for elliptic equations involving limiting nonlinearities. Proc. Jpn. Acad. Ser. A..

[22] Anastassiou GA (2011). Multivariate Lyapunov inequalities. Appl. Math. Lett..

[23] de Nápoli, PL, Pinasco, JP: Lyapunov-type inequalities for partial differential equations. arXiv:1304.6988 [math.AP]

[24] de Nápoli PL, Pinasco JP (2006). Estimates for eigenvalues of quasilinear elliptic systems. J. Differ. Equ..

